# Goal-directed hemostatic therapy using rotational thromboelastometry in patients requiring emergent cardiovascular surgery

**DOI:** 10.1186/cc13281

**Published:** 2014-03-17

**Authors:** D Sartorius, JL Waeber, G Pavlovic, M Aldenkortt, MJ Licker

**Affiliations:** 1 Hôpital Cantonal Universitaire de Genève, Geneva, Switzerland Critical Care

## Introduction

Massive bleeding remains a leading cause of potentially preventable death after cardiovascular surgery [1]. Conventional coagulation tests (CCT) fail to characterize the multiple hemostatic abnormalities observed in surgical patients and are further limited by their slow results and poor correlation with transfusion requirements. We assessed the clinical impact of goal-directed coagulation management based on rotational thromboelastometry (ROTEM) in patients undergoing an emergent cardiovascular surgical procedure.

## Methods

Over a 2-year period, data from 71 patients were collected prospectively and blood samples were obtained for coagulation testing. Administration of packed red blood cells (PRBC) and hemostatic products was guided by an algorithm using ROTEM- derived information and hemoglobin level. Based on the amount of PRBC transfused, two groups were considered: high bleeders (≥5 PRBC; HB) and low bleeders (<5 PRBC; LB). Data were analyzed using the chi- square test, unpaired *t *test and ANOVA as appropriate.

## Results

Preoperatively, the HB group (*n *= 31) was characterized by lower blood fibrinogen and decreased clot amplitude at ROTEM compared with the LB group (*n *= 40). Intraoperatively, larger amounts of fibrinogen, fresh frozen plasma and platelets were deemed necessary to normalize the coagulation parameters in the HB group. Postoperatively, the incidence of major thromboembolic and ischemic events did not differ between the two groups (<10%) and the observed in-hospital mortality was significantly less than expected by the POSSUM score (22% vs. 35% in HB group and 5% vs. 13% in LB group).

## Conclusion

ROTEM-derived information is helpful to detect early coagulation abnormalities and to monitor the response to hemostatic therapy. Early goal-directed management of coagulopathy may contribute to improve outcome after cardiovascular surgery.
